# One-Pot Facile Methodology to Synthesize Chitosan-ZnO-Graphene Oxide Hybrid Composites for Better Dye Adsorption and Antibacterial Activity

**DOI:** 10.3390/nano7110363

**Published:** 2017-11-02

**Authors:** Anandhavelu Sanmugam, Dhanasekaran Vikraman, Hui Joon Park, Hyun-Seok Kim

**Affiliations:** 1 Department of Chemistry (S&H), Vel Tech Multitech Dr.Rangarajan Dr.Sakunthala Engineering College, Chennai 600062, India; sranand2204@gmail.com; 2 Division of Electronics and Electrical Engineering, Dongguk University-Seoul, Seoul 04620, Korea; 3 Department of Electrical and Computer Engineering, Ajou University, Suwon 16499, Korea; huijoon@ajou.ac.kr; 4 Department of Energy Systems Research, Ajou University, Suwon 16499, Korea

**Keywords:** nano hybrid composites, FTIR, chitosan, dye adsorption, TEM, antibacterial activity

## Abstract

Novel chitosan–ZnO–graphene oxide hybrid composites were prepared using a one-pot chemical strategy, and their dye adsorption characteristics and antibacterial activity were demonstrated. The prepared chitosan and the hybrids such as chitosan–ZnO and chitosan–ZnO–graphene oxide were characterized by UV-Vis absorption spectroscopy, X-ray diffraction, Fourier transform infrared spectroscopy, scanning electron microscopy, and transmission electron microscopy. The thermal and mechanical properties indicate a significant improvement over chitosan in the hybrid composites. Dye adsorption experiments were carried out using methylene blue and chromium complex as model pollutants with the function of dye concentration. The antibacterial properties of chitosan and the hybrids were tested against Gram-positive and Gram-negative bacterial species, which revealed minimum inhibitory concentrations (MICs) of 0.1 µg/mL.

## 1. Introduction

The advancement of nanotechnology has led to a variety of nanomaterials that require investigations into their safety for human health and ecological purposes at the environmental and organism levels [1]. Many research groups have paid attention to developing various types of antimicrobial agents and novel materials to protect human life against the negative effects of microorganisms [2,3,4], and in particular, targeting pathogenic bacteria with nanomaterials has received great attention [5,6]. Despite their importance, it is crucial for antimicrobial agents to be able to pass through the cell membrane and show a very low level of activity in cells [7]. Similarly, dyes can be harmful to flora and fauna with some organic dyes and their by-products having a mutagenic or carcinogenic effect in human beings [8,9,10] as well as causing allergic dermatitis and skin irritation [11]. Adsorptive removal is the most widely used method for various dyes because of the ease of operation and compatibility in low cost applications [12,13,14]. Methylene blue (MB) and chromium complex (CC) are the most commonly used substances for dyeing cotton, wool, and silk, and exposure to them may cause nausea, vomiting, profuse sweating, mental confusion, and methemoglobinemia [15,16]. Therefore, the removal of MB and CC from waste effluents is environmentally important.

Chitosan (CS), a copolymer of β[1,4]-linked 2-acetamido-2-deoxy-d-glucopyranose and 2-amino-2-deoxy-d-glucopyranose and one of the most plentiful natural polymers on earth, is generally obtained through deacetylation of chitin [17]. Due to its biodegradability, biocompatibility, and lack of toxicity, it has been used in a significantly broad range of applications in different fields such as the biomedical, food, water treatment, membrane separation, textile, and paper industries [18]. There have been a few reports based on silver nanoparticles, metal oxides, and graphene oxides used as antimicrobial agents with CS [19,20,21]. As a well-known sorbent, CS is widely used for the removal of heavy metals and dyes [22,23,24]. However, it can only adsorb very small amounts of cationic dyes because it is a natural cationic polysaccharide. Moreover, the relatively high market cost and low specific gravity also limit its practical use. Therefore, several efforts have been made to develop more effective adsorbents. Zinc oxide (ZnO) is a versatile semiconductor material with a wide bandgap of ~3.37 eV and large excitation binding energy (60 mV) at room temperature [25,26,27,28,29]. ZnO is recognized as a safe material, and it has the inherent advantage as a broad antibacterial activity material against fungi, viruses, and bacteria [30,31,32,33,34]. At present, developing ZnO nanoparticles with excellent antibacterial properties and less toxicity to other species is still an attractive challenge. The antibacterial behavior of nanomaterials has mostly emerged due to their high specific surface area-to-volume ratios [35] and unique physicochemical properties [36,37]. Moreover, ZnO particles are easily agglomerated by coalescence, which is able to decrease aggregation with an organic reagent or stable polymer [37,38].

Graphene oxide (GO) is an oxidized derivative of graphene, a fascinating carbon material that has attracted strong attention because of its promising ability to adsorb dyes and supporting catalysts due to its superior mechanical strength, relatively large specific area [39], and good biocompatibility [40]. Graphene-based materials have also shown excellent antibacterial activity because of their mechanical strength and high thermal stability; e.g., the resection of GO within sheets is a mechanism that inactivates bacteria [41,42,43]. Thus, it is of interest to researchers to explore novel hybrid materials with different physical and chemical compositions in order to increase antibacterial activity. Effective modification of GO would prevent the aggregation of ZnO particles and result in strong stability in an ambient environment [44]. Based on the favorable adsorption properties of CS and the inherent properties of GO, some research groups have reported CS-GO composites as bioadsorbents [45,46].

In this work, we used a one-pot chemical strategy to synthesize CS and chitosan–ZnO (CS–ZnO) and chitosan–ZnO–graphene oxide (CS–ZnO–GO) hybrids. Interestingly, we discovered that the CS-ZnO-GO hybrid exhibited strong antibacterial activity against *E. coli* and *S. aureus* and good dye adsorption behavior for MB and CC. To the best of our knowledge, there have been no reports published on dye adsorption and antibacterial studies for hybrid composites made from a combination of CS, GO, and ZnO.

## 2. Results

**Figure 1:**
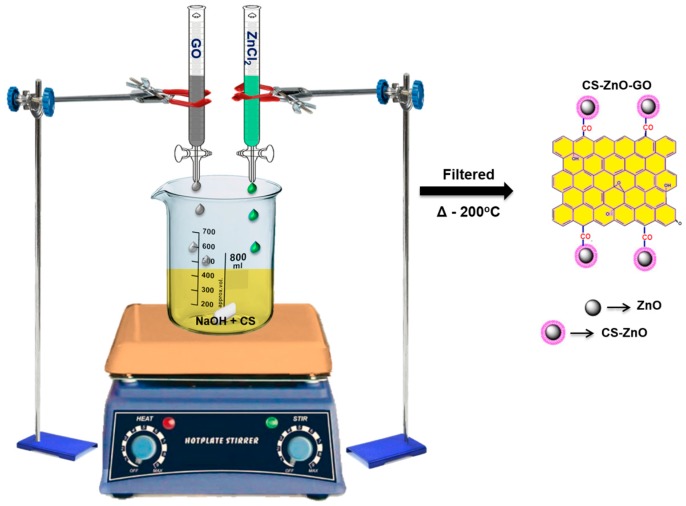
Schematic diagram for chitosan–ZnO–graphene oxide (CS–ZnO–GO) hybrid composite preparation.

**Figure 2:**
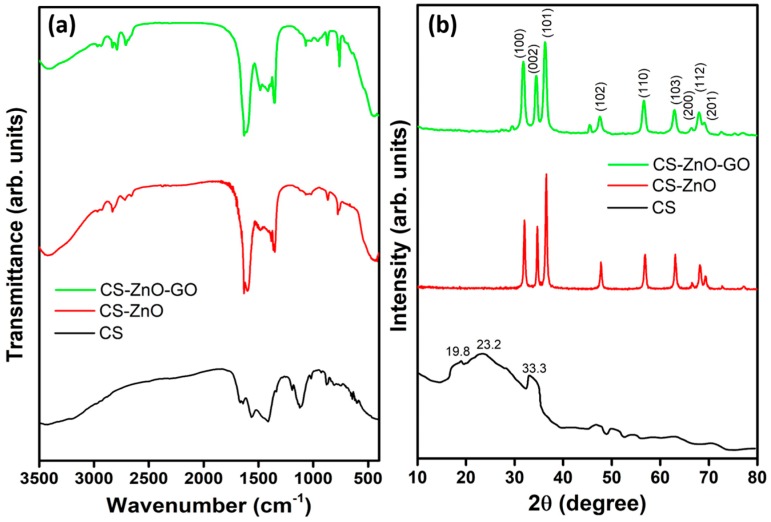
(**a**) Fourier transform infrared (FTIR) and (**b**) X-ray diffraction (XRD) spectra of CS and the CS–ZnO and CS–ZnO–GO hybrid structures.

We successfully established the synthesis of CS and the CS–ZnO and CS–ZnO–GO hybrids using a one-pot chemical strategy, a schematic representation of which is given in Figure 1. Fourier transform infrared (FTIR) spectral analyses were carried out to confirm the formation of CS and the hybrid nanocomposites, as shown in Figure 2a. In the FTIR spectrum of the CS sample, the stretching vibration of the O–H functional group appeared at 3438 cm^−1^. In addition, there were two characteristic bands centered at 1651 and 1571 cm^−1^ corresponding to the C=O stretching vibration of –NHCO– and the N–H bending of –NH_2_, respectively [47]. Transmittance peaks were observed at 1641 and 1411 cm^−1^ corresponding to the C=C vibration and O–H bending, respectively [48,49]. The intense peak occurring at 1107 cm^−1^ is due to C–O–C stretching with a shoulder peak of anti-symmetric stretching of the (C–O–C) bridge at 1195 cm^−1^ [47]. Moreover, bands at 1016 and 873 cm^−1^ were derived from skeletal vibration involving C–O stretching and out-of-plane O–H, respectively [50]. The detailed peak positions and their functional groups for the CS sample are provided in supporting information Table S1.

For the CS–ZnO and CS–ZnO–GO samples, the FTIR curves exhibited ZnO and GO related peaks in addition to the CS sample peaks. Functional groups such as N–H bending of the primary amine (@ ~2967 cm^−1^), C–O–C stretching (@ 2928 cm^−1^) and alkyl stretching (@ ~2834, ~2726, and ~2654 cm^−1^) were observed for both the CS–ZnO and CS–ZnO–GO samples [50,51]. The peaks at ~1631 and ~1348 cm^−1^ were due to the carbonyl group interacting with the Zn atom of the ZnO and O–H deformation of the C–OH groups, respectively [52]. A FTIR peak was observed at 1492 cm^−1^ for CS–ZnO attributed to the bond formation of the COO– group with ZnO, which was shifted to 1484 cm^−1^ for CS–ZnO–GO [53]. Due to the incorporation of GO by CS–ZnO, C–H bending vibration (@1413 cm^−1^), C–O–C stretching vibration (@ 1071 cm^−1^), and C–O stretch (@ 953 cm^−1^) functional groups were observed for the CS–ZnO–GO hybrid. For CS–ZnO, a characteristic peak of stretching mode vibration appeared at ~440 cm^−1^ for the confirmation of Zn–O bond formation [48]. In the FTIR spectrum of CS–ZnO–GO, the characteristic Zn–O stretching vibration frequency was shifted to a higher wave number (462 cm^−1^), which might have been due to the carboxylic functional groups involved in the formation of Zn–O–C [43,54]. Furthermore, this might have been due to the contribution of carboxylic functional groups in the formation of Zn–O–C carbonaceous bonds for the CS–ZnO–GO functionalized hybrid composite [54,55]. The detailed peak positions and their functional groups for CS-ZnO and CS-ZnO-GO are provided in supporting information Tables S2 and S3, respectively.

Furthermore, structural confirmation studies were carried out using X-ray diffraction (XRD) analysis. Figure 2b shows the XRD patterns of CS and the CS–ZnO and CS–ZnO–GO composites. The CS-based 2θ peaks were observed at 19.8, 23.2, and 33.3. The predominant peak orientation of the (101) lattice plane was observed for CS–ZnO and CS–ZnO–GO composites, and the observed peaks were indexed with a standard hexagonal structure (JCPDS-36-1451). In addition, other diffraction lines related to the (100), (002), (102), (110), (103), (200), (112), and (201) planes of the lattice orientation of ZnO were observed for the CS–ZnO and CS–ZnO–GO samples. Peak broadening decreased more with intensity for CS–ZnO–GO than CS–ZnO, which is attributed to the incorporation of GO into the CS lattice in the former. In addition, the CS peak vanished due to the higher crystalline properties of ZnO. Furthermore, we estimated the crystallite size of the nanocomposites using Debye-Scherer’s formula to help deduce their microstructural characteristics [56,57]. Consequently, the crystallite sizes for the CS–ZnO and CS–ZnO–GO hybrids were estimated as 23.2 and 19.5 nm, respectively.

**Figure 3:**
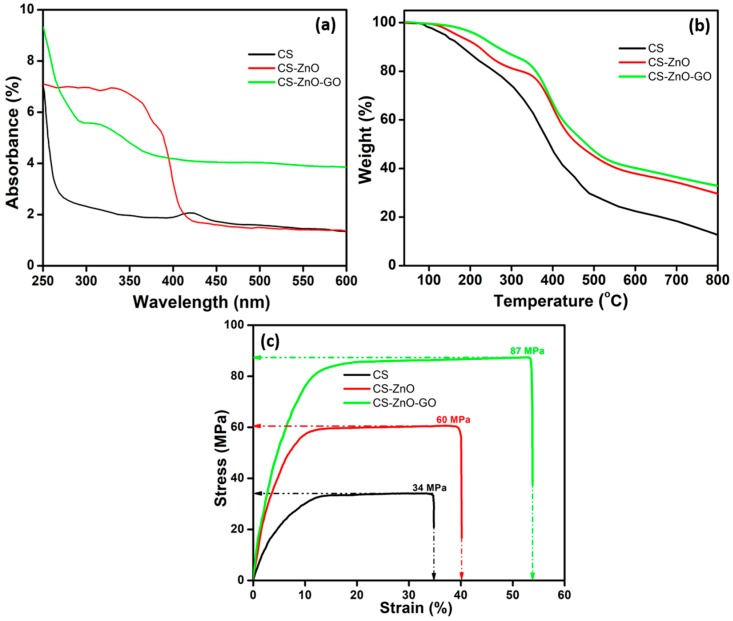
(**a**) UV–Vis spectra; (**b**) thermogravimetric (TGA) curves; and (**c**) mechanical properties of CS and the CS–ZnO and CS–ZnO–GO hybrid structures.

UV–Vis absorption spectra of CS, CS–ZnO, and CS–ZnO–GO samples are shown in Figure 3a. For the CS sample, an absorption band edge was observed at around 260 nm, which was mainly due to the transition of its amino groups from *n*→*σ*∗ and the presence of chromophores [58]. The adsorption band observed at around 420 nm might have been due to characteristic behavior of CS [59]. ZnO was dominant in optical absorption behavior of CS–ZnO sample and the band edge shifted to ~400 nm, which is highly consistent with earlier results. After combining GO with CS and ZnO, an absorption band edge shifted toward the blue region at around 290 nm and also absorption decreased slightly, which suggests the successful formation of CS with ZnO and GO hybrid nanocomposites [51,60]. The thermal properties of the hybrid composites were determined by thermogravimetric analysis (TGA). TGA curves for CS and the CS–ZnO and CS–ZnO–GO hybrids are provided in Figure 3b. From the TGA curve of the CS sample, weight loss of less than 5% up to 100 °C was observed, which might have been due to the volatilization of free and hydrogen bonded water. Thereafter, rapid weight loss was observed until 480 °C, which was attributed to the decomposition of CS, and the sample had a residual weight of 13% at 800 °C. For the CS–ZnO and CS–ZnO–GO samples, the rate of decomposition was decreased effectively and the peak observed at around at 350 °C was due to ZnO [61]. The tremendous improvement in thermostability in the CS–ZnO–GO hybrid can be explained by the existence of strong interactions of the ZnO nanomaterial with CS and GO. The presence of the GO structure within the matrix system was also able to act as a thermal barrier, leading to improved thermal stability [52].

The stress-strain profiles generated by tensile testing indicate the mechanical behavior of the pure CS matrix as well as the CS–ZnO and CS–ZnO–GO nanocomposites. The typical stress-strain curves of CS and the CS–ZnO and CS–ZnO–GO nanocomposites are shown in Figure 3c. For the CS sample, the stress-strain profile shows two discrete regions: a linear region for elastic characteristic and a nonlinear region for plastic deformation. The tensile strength was 34 MPa while the strain was 35%. In the case of the CS–ZnO and CS–ZnO–GO samples, the mechanical strength and flexibility improved linearly. For CS–ZnO–GO, the tensile strength increased sharply to 87 MPa while the strain increased to 54% (Figure 3c). Furthermore, it is interesting to note that the CS–ZnO–GO nanocomposite had a higher tensile strength in addition to increased elongation compared to pure CS and CS–ZnO, which is dissimilar behavior to other GO-based nanocomposites such as poly(vinyl alcohol)/GO [62] and CS/carbon nanotubes [63,64]. Nevertheless, in some cases, simultaneous improvement of tensile strength and elongation of polymer nanocomposites through the incorporation of oriented or functionalized nanofillers [65,66] and carbon nanotube-based nanocomposites [67,68] have been reported. In general, good dispersion and interfacial stress transfer are important factors for preparation of reinforcing nanocomposites. This leads to a more uniform stress distribution and minimizes the presence of the stress concentration center [69]. The compatibility and strong interaction between GO, ZnO, and the CS matrix was greatly enhanced by the unidirectional dispersion of GO and ZnO within the CS matrix on the molecular scale as well as interfacial adhesion, thus significantly increasing the mechanical properties of the nanocomposites.

**Figure 4:**
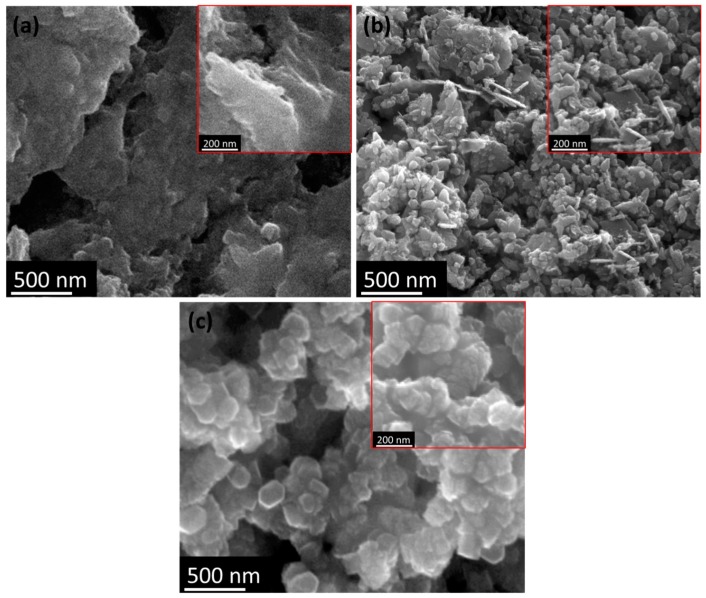
Scanning electron microscopy (SEM) images of (**a**) CS and the (**b**) CS–ZnO and (**c**) CS–ZnO–GO hybrid structures.

**Figure 5:**
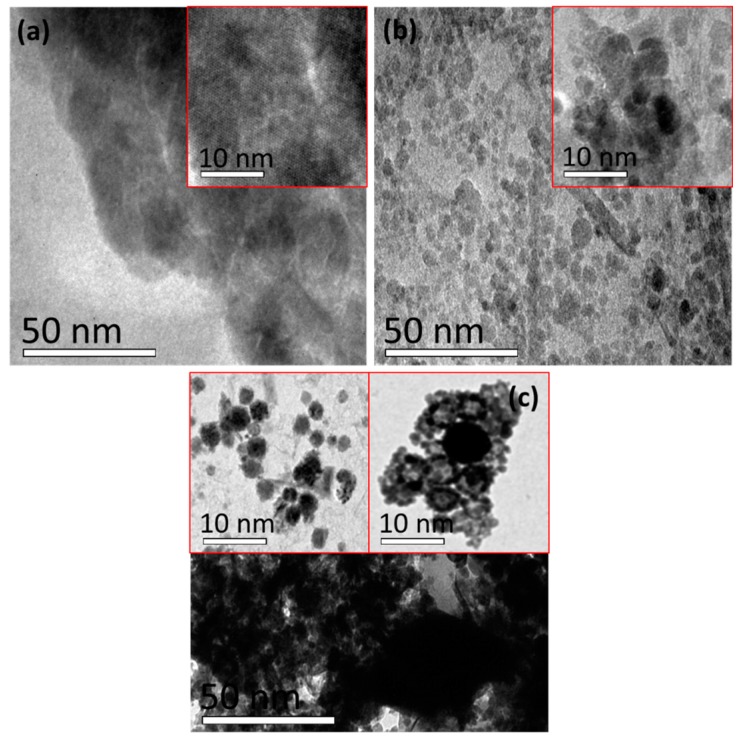
Transmission electron microscopy (TEM) images of (**a**) CS and the (**b**) CS–ZnO and (**c**) CS–ZnO–GO hybrid structures (Inset—corresponding higher magnification TEM images).

To demonstrate their morphological properties, scanning electron microscopy (SEM) images of different hybrid composites are shown in Figure 4a–c. Figure 4a shows the amorphous nature of the surface due to the semi-crystalline behavior of CS, as previously demonstrated in the XRD analysis (Figure 2b). Rod- and cuboid-shaped grains were observed after ZnO was introduced into the CS matrix (Figure 4b), which were a larger size than CS due to the agglomeration process. Hillock-shape morphology with voids exhibited in the CS–ZnO–GO hybrid composite was due to agglomeration, as shown in Figure 4c. From the SEM images, GO and ZnO enhanced the agglomeration process with CS to form strongly bonded hybrid composites. Furthermore, the size of the grains for CS and the CS–ZnO and CS–ZnO–GO hybrid nanocomposites was analyzed using transmission electron microscopy (TEM), as shown in Figure 5. Amorphous background nanoparticles were confirmed in the TEM image of the CS sample (Figure 5a). For the CS–ZnO hybrid (Figure 5b), the rod- and cuboid-shaped grains were clearly elucidated with the sizes of the grains being in the range of ~5–15 nm. Moreover, the grain bunches of ~5–10 nm size were evidently demonstrated for the CS–ZnO–GO sample, as shown in Figure 5c. The TEM surface profile spectra of CS and the CS–ZnO and CS–ZnO–GO hybrids are provided in supporting information Figures S5–S7, which clearly indicate that our prepared hybrids consisted of nanosized grains.

**Figure 6:**
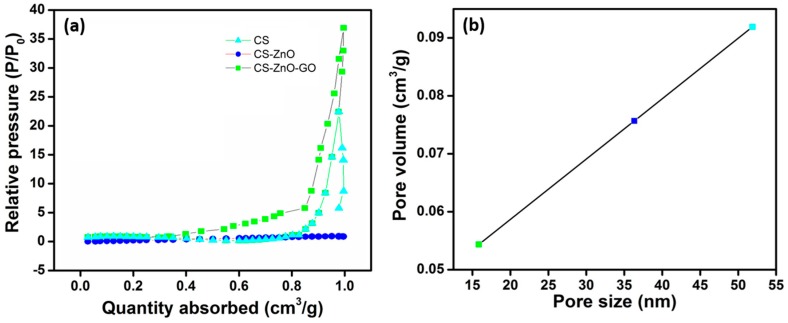
(**a**) Nitrogen adsorption–desorption isotherms and (**b**) pore volume versus pore size distribution of CS and the CS–ZnO and CS–ZnO–GO hybrid structures.

The specific surface area and pore size distribution of CS and the CS–ZnO and CS–ZnO–GO hybrids were characterized using nitrogen (N_2_) gas sorption. The N_2_ adsorption–desorption isotherms showed a typical international union of pure and applied chemistry (IUPAC) type IV characteristics with distinct hysteresis loops at relative pressures of 0.5–1.0 P/P_0_ ca (Figure 6a). The specific surface area of the CS–ZnO–GO hybrid was evaluated at 38.2 m^2^/g, but the observed specific surface area of CS (22.5 m^2^/g) was much smaller [55]. The observed pore volume values were 0.076, 0.057, 0.098 cm^3^/g for CS, CS–ZnO, and CS–ZnO–GO, respectively. The measured pore volume for CS–ZnO–GO (0.098 cm^3^/g) was almost double that of CS–ZnO (0.057 cm^3^/g). The variations of pore size against pore volume (Figure 6b) indicate that the CS–ZnO–GO sample had the highest porous structure with an average pore radius of ~52 nm. This evidence supports the enhancement of the surface area of CS–ZnO–GO, leading to good sorption ability.

**Figure 7:**
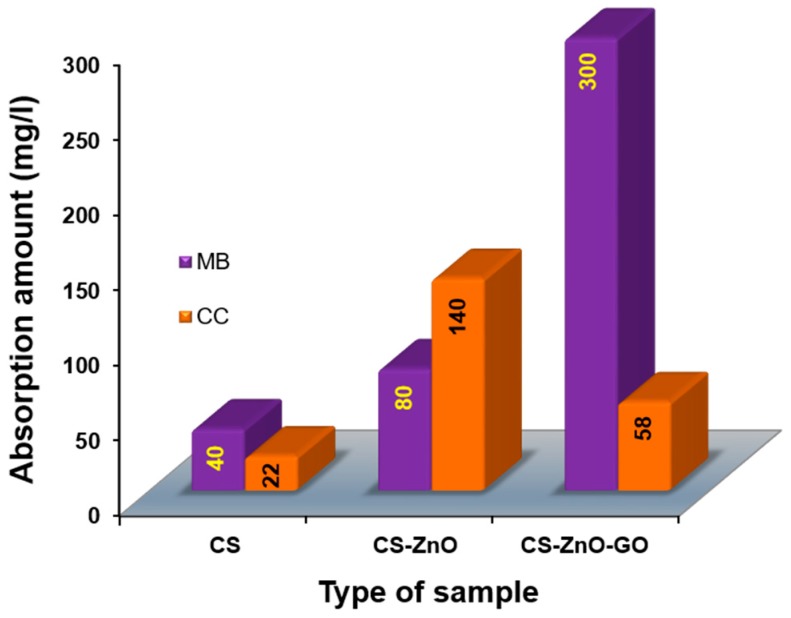
Comparison of adsorption amounts of methylene blue (MB) and chromium complex (CC) dyes by CS and the hybrid structures with a contact time of 20 min.

The adsorption behavior of CS, CS–ZnO, and CS–ZnO–GO for methylene blue (MB) and chromium complex (CC) dyes as model pollutants are shown in Figure 7. The absorption amount increased rapidly for CS–ZnO–GO, which was due to the higher number of carboxylic and oxygenated functional groups in GO. The adsorbed amounts of MB dye (Q) were 40, 80, and 300 mg/L whereas adsorbed amounts of CC dye (Q) were at 22, 140, and 58 mg/L for CS, CS–ZnO, and CS–ZnO–GO, respectively. For example, Neumann et al. [70] reported that after photocatalysis by TiO_2_–graphene composites, a considerable amount of MB remained in solution (2 mg/L). Because it was able to decolorize MB solution over a wide concentration range, CS–ZnO–GO hybrid composite might be applicable to treating not only industrial effluent but also contaminated natural water. CS–ZnO showed the best absorption CC dye, and CS–ZnO–GO showed the best absorption of MB dye. Compared to the other approaches, our CS–ZnO–GO hybrid performed the best even with very low MB concentration, which makes it feasible for use with industrial effluent.

**Figure 8:**
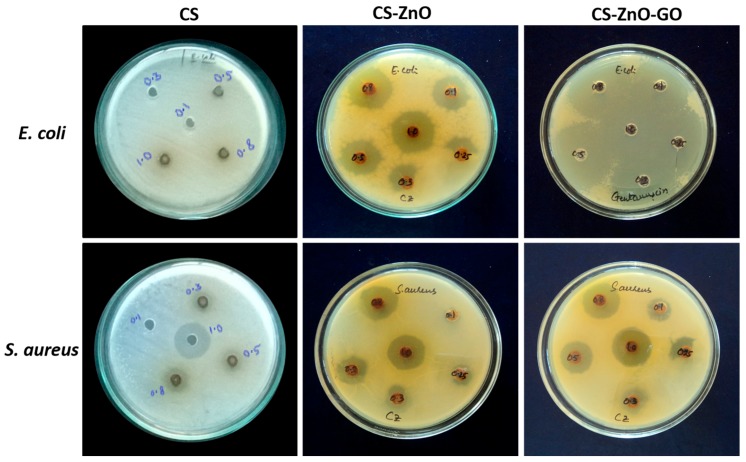
Antibacterial studies with CS and the CS–ZnO and CS–ZnO–GO hybrids against *E. coli* and *S. aureus.* The samples were incubated at 35 °C for 24 h.

Antibacterial studies for each of the test samples against *Staphylococcus aureus* (*S. aureus*) and *Escherichia coli* (*E. coli*) are exhibited in Figure 8. To each zone, 100 µL of a solution of each at different concentrations (0.1, 0.3, 0.5, 0.8, and 1.0 µg/mL) was added and the obvious inhibition zones were measured in the agar plates after incubation, as shown in Figure 8; the minimum inhibitory concentrations (MICs) against *E. coli* and *S. aureus* are tabulated in Table 1. Our composite samples were found to have superior antibacterial effects as they were able to kill *S. aureus* and *E. coli*, known respectively to be the most resistant Gram positive [71] and Gram negative [72] bacteria, and to be responsible for infections in wounds and contamination of foodstuffs [53]. We found that CS–ZnO–GO and CS–ZnO were able to inhibit the bacterial growth at lower concentrations than CS. The zone of inhibition values for different concentrations of the CS–ZnO–GO hybrid against *S. aureus* and *E. coli* are provided in supporting information Table S4.

The observed results confirmed that the symbiotic effect of CS, ZnO, and graphene oxide was responsible for the strong anti-bacterial efficiency [43,55]. From earlier reports of antibacterial activity using various nanoparticles, oxidative stress is a highly recognized mechanism [41,73,74,75,76]. GO is a special two-dimensional structure that can interact strongly with the bacterial lipid bilayer, which causes lipid molecules to separate from the membrane and attach to GO sheets, thereby resulting in destruction of the bacterial membrane [30,73]. In an earlier study, the structural and physiochemical properties of carbon nanomaterials induced oxidative stress, which is a key antibacterial mechanism [74]. CS is a cationic polysaccharide derived from chitin that has a positive surface charge able to attract the negatively charged cell membrane of bacteria, which was enhanced by the interaction between CS, ZnO, and/or GO in the nanocomposites [51]. In addition, earlier reports illustrated that ZnO induces reactive oxygen species (ROS) dependent on oxidative stress, which kill the bacteria [76]. Moreover, electrons can rapidly transfer between ZnO and GO in the composite, absorbing surface oxygen to form various ROS and ultimately leading to the formation of lipid peroxide that is able to damage the bacterial membrane. The antibacterial activity of CS–ZnO–GO is attributed to the production of ROS, including singlet oxygen, superoxide ions, and hydroxyl radicals [73]. In an earlier study, the antimicrobial activity in Ag/GO suspensions against *S. aureus* and *E. coli* illustrated the higher importance of Ag nanoparticles compared to GO for strong antibacterial activity [54]. Our observed results suggest that a synergistic effect between CS, ZnO, and GO in the CS–ZnO–GO hybrid caused complete bacterial inhibition [75,77], and we envisage that this study offers novel insights into its antimicrobial action while also demonstrating that CS–ZnO–GO is a novel class of topical antibacterial agent useful in the areas of healthcare and environmental engineering.

## 3. Materials and Methods

### 3.1. Materials

Deionized (DI) water was used to prepare all of the experimental solutions. Sulfuric acid (H_2_SO_4_), potassium permanganate (K_2_MnO_4_), hydrogen peroxide (H_2_O_2_), zinc chloride (ZnCl_2_), HCl, acetic acid (CH_3_COOH), and NaOH were obtained from Sigma-Aldrich chemicals (Sigma-Aldrich, Mumbai, India). For chitin preparation, the collected crumbs of crab shells were washed, dehydrated, and powdered and then treated by demineralization and deproteinization processes separately using hydrochloric acid (HCl) and sodium hydroxide (NaOH) solutions, respectively, for 120 min. Commercially available graphite powder was purchased from Loba Chemie chemicals (Loba Chemie Pvt. Ltd, Mumbai, India). GO solution was synthesized from graphite powder using a modified Hummers and Offeman procedure [78,79]. 

### 3.2. Synthesis of Hybrid Composites

At the beginning, extracted chitin (0.25 g) was dissolved in CH_3_COOH and subjected to constant magnetic stirring for 2 h at 100 °C bath temperature to obtain a pale yellow chitin solution. Thereafter, a freshly prepared (45%) NaOH solution was microadded until the formation of a white colored CS precipitate that settled at the bottom of the flask, a process that took up to 24 h. Finally, the precipitate was filtered using a suction pump and dried in a hot air oven at 200 °C [60]. For the CS–ZnO composite, 15% ZnCl_2_ solution was added dropwise into the pale yellow chitin solution and then precipitated by the microaddition of NaOH solution. For the CS–ZnO–GO hybrid composite preparation, 15% zinc chloride solution and 20 mL of as-prepared GO solution were added one-by-one dropwise to the pale yellow chitin solution and then precipitated by microaddition of NaOH solution. The prepared hybrid composites were soluble in water at acidic pH (~2 ± 0.1).

### 3.3. Characterization

FTIR spectra were recorded using a Thermo-Nicolet-380 model (Thermo Fisher, Madison, WI, USA) spectrum in the range of 3500–400 cm^−1^ at room temperature. Structural studies were performed using an X-ray diffractometer (X’Pert PRO PANalytical diffractometer, Almelo, The Netherlands) with CuK_α_ radiation (λ = 0.154 nm). Absorption spectra were recorded using a UV–Vis spectrophotometer (2401 PC model; Shimadzu, Kyoto, Japan) in the wavelength range of 250–600 nm. The mechanical stability of our hybrids were measured with an Instron Tester 6025. The surface area and porosity were determined from N_2_ adsorption/desorption isotherms with a Micromeritics ASAP 2020 physisorption instrument (Micromeritics, Norcross, GA, USA) using the BET equation to estimate the overall surface area. Morphological properties were analyzed using a scanning electron microscope (model Hitachi-S3000 H, Hitachi, Tokyo, Japan). The size of hybrid structures was observed using a Philips CM200 transmission electron microscope with an accelerating voltage of 200 keV (FEI, Hillsboro, OR, USA). Image processing (surface profile) was performed using Gatan Digital Micrograph software (Gatan Microscopy Suite 3.0).

### 3.4. Dye Absorption and Antibacterial Activity

The standardization curve of UV–Vis spectra for MB and CC dyes with their structure (inset) is provided in Figures S1 and S2. The standardisation study was performed with different concentrations of dye solution: 15, 30, 45, and 60 mg/L. The absorbance spectra were recorded using a UV–Visible spectrophotometer. Five tests for each dye were recorded and their average values of absorption intensity were measured (absorbance λ_max_ at 620 nm for MB and CC λ_max_ at 579 nm). The linear plots of absorption intensity against dye concentration are shown in Figures S3 and S4.

The adsorption experiments were performed using a thermostat shaker with a shaking speed of 180 rpm. Typically, a 10 mL solution of 60 mg concentration MB and CC dyes was added separately into 100 mL glass flasks and then shaken at 30 ± 0.2 °C. Subsequently, 10 mL of solution containing 0.05 g adsorbents was added with a contact time of 20 min. Residual MB and CC concentration in the supernatant was determined using dye adsorption experiments with a UV–Visible spectrophotometer. The adsorption amount of the MB or CC concentration in the aqueous solution adsorption was calculated according to the following equation:

*Q* = (*C*_0_ − *C_e_*) *V*/*W* (1)

where *C*_0_ and *C_e_* are the initial and equilibrium concentrations of MB or CC in mg/L, respectively; *V* is the volume of MB or CC solution in L; and *W* is the weight of the CS, CS–ZnO, or CS–ZnO–GO used in mg.

The antibacterial activity of the nano composites was screened against *E. coli* (ATCC 25922) and *S. aureus* (ATCC 25923). The bacteria were cultured overnight at 35 °C, and then the cultures were centrifuged at 5000 rpm for 15 min. Afterwards, the pallets were washed with sterile phosphate buffered saline (PBS). Broths containing 100 μL of CS, CS–ZnO, or CS–ZnO–GO solution were prepared at different concentrations and then microwell agar plates were inoculated with the bacterial inoculum. The plates were incubated at 35 °C for 24 h. The final concentration of the inoculum was 10^6^ colony forming units (CFU) per ml of broth. Absorbance in the microwell plates was measured at 620 nm using a UV spectrophotometer (2401 PC model; Shimadzu, Kyoto, Japan) to evaluate MIC values.

## 4. Conclusions

In summary, CS, CS–ZnO, and CS–ZnO–GO acted as good adsorbents of MB and CC dyes in aqueous solutions and their batch adsorption experiments were investigated in detail. The synergistic effect between CS, ZnO, and GO was evident in the antibacterial analysis, in which CS–ZnO–GO completely inhibited the growth of *E. coli* and *S. aureus*. The observed results revealed that the CS–ZnO–GO hybrid composite is a promising solution for inhibiting bacteria propagation and absorbing toxic dyes in cases of water treatment, food packaging, adhesives, tissue engineering, medical, and pharmaceutical applications.

## Figures and Tables

**Table 1:** Minimum inhibitory concentration (MIC) values of chitosan (CS) and its hybrids against *E. coli* and *S. aureus.*

Bacteria	MIC of CS (µg/mL)	MIC of CS–ZnO (µg/mL)	MIC of CS–ZnO–GO (µg/mL)
*E. coli*	0.5	0.1	0.1
*S. aureus*	0.3	0.1	0.1

## Supplementary Materials

The following are available online at http://www.mdpi.com/2079-4991/7/11/363/s1. Table S1: FTIR peaks and their functional groups for the CS sample, Table S2: FTIR peaks and their functional groups for the CS–ZnO sample, Table S3: FTIR peaks and their functional groups for the CS–ZnO–GO sample, Table S4: Zone of inhibition for the CS–ZnO–GO sample against *E. coli* and *S. aureus*, Figure S1: UV–Vis calibration curves of methylene blue (inset—methylene blue chemical structure), Figure S2: UV–Vis calibration curves of chromium complex (inset—chromium complex chemical structure), Figure S3: Variation in absorbance with dye concentration for methylene blue, Figure S4: Variation in absorbance with dye concentration for chromium complex, Figure S5: TEM surface profile spectrum of the CS sample, Figure S6: TEM surface profile spectrum of the CS–ZnO hybrid structure, Figure S7: TEM surface profile spectrum of the CS–ZnO–GO hybrid structure.
