# Supplementary material for: One-Pot Facile Methodology to Synthesize Chitosan-ZnO-Graphene Oxide Hybrid Composites for Better Dye Adsorption and Antibacterial Activity
Source: Nanomaterials (Basel). 2017 Nov 2;7(11):363. doi: 10.3390/nano7110363 (PMC5707580; doi:10.3390/nano7110363)
Supplement: Supplementary file 1 [file nanomaterials-07-00363-s001.pdf]

One-Pot Facile Methodology to Synthesize Chitosan-ZnO-Graphene Oxide Hybrid Composites for Better Dye Adsorption and Antibacterial Activity

Anandhavelu Sanmugam [1], Dhanasekaran Vikraman [2,*], Hui Joon Park [3,4] and Hyun-Seok Kim [2,*]

Electronic Supporting Information

Table S1. FTIR peaks and their functional groups for the CS sample

**Wavenumber (cm^{-1})**	**Functional groups**
3442	Stretching vibration of O-H
1666	C═O stretching vibration of −NHCO
1641	C=C vibration
1571	N−H bending of $-NH_2$
1411	O-H bending
1195	anti- symmetric stretching of (C–O–C) bridge
1107	C-O-C stretching
1016	skeletal vibration involving C–O stretching
873	O-H out-of-plane

Table S2. FTIR peaks and their functional groups for the CS-ZnO sample

**Wavenumber (cm^{-1})**	**Functional groups**
3421	Stretching vibration of O-H
2967	N–H bending of primary amine
2928	C–O–C stretching

2834	alkyl stretching
2726	alkyl stretching
2654	alkyl stretching
1631	Carbonyl group interacting with the Zn atom of the ZnO
1591	N–H bending of $-NH_2$
1492	COO− group with ZnO
1348	O-H deformations of C-OH groups
1016	skeletal vibration involving C–O stretching
863	O-H out-of-plane
440	Zn-O

Table S3. FTIR peaks and their functional groups for the CS-ZnO-GO sample

Wavenumber (cm^{-1})	Functional groups
3417	Stretching vibration of O-H
2968	N–H bending of primary amine
2932	C–O–C stretching
2828	alkyl stretching
2791	alkyl stretching
2707	alkyl stretching
1629	Carbonyl group interacting with the Zn atom of the ZnO

1484	COO− group with ZnO
1413	C-H bending
1356	O-H deformations of C-OH groups
1071	C–O–C stretching
953	C-O stretch
874	O-H out-of-plane
462	Zn-O

Table S4. Zone of inhibition for the CS-ZnO-GO sample against *E. coli* and *S. aureus*

S.No	Bacteria	Zone of inhibition (mm in diameter)				
		0.1 μg/mL	0.3 μg/mL	0.5 μg/mL	0.8 μg/mL	1.0 μg/mL
1	*E.coli*	23	25	27	29	30
2	*S. aureus*	16	17	19	22	24

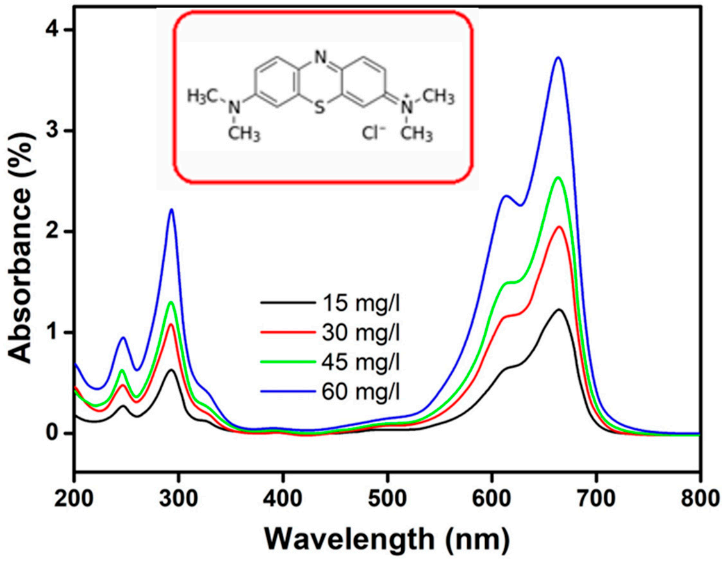

Figure S1. UV-Vis calibration curves of methylene blue (inset-methylene blue chemical structure)

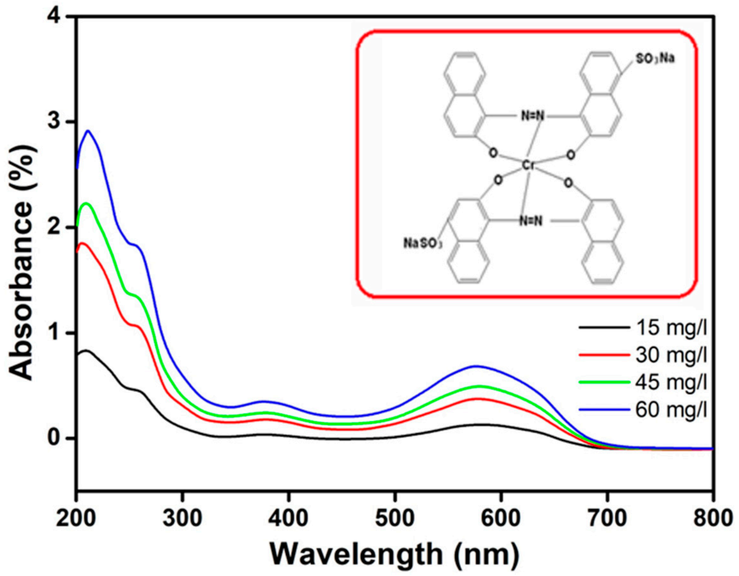

Figure S2. UV-Vis calibration curves of chromium complex (inset-chromium complex chemical structure)

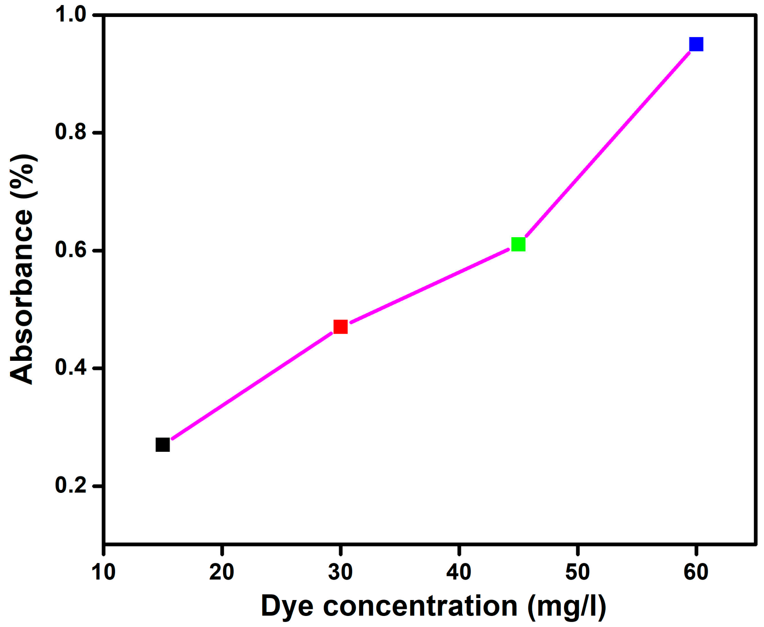

Figure S3. Variation in absorbance with dye concentration for methylene blue

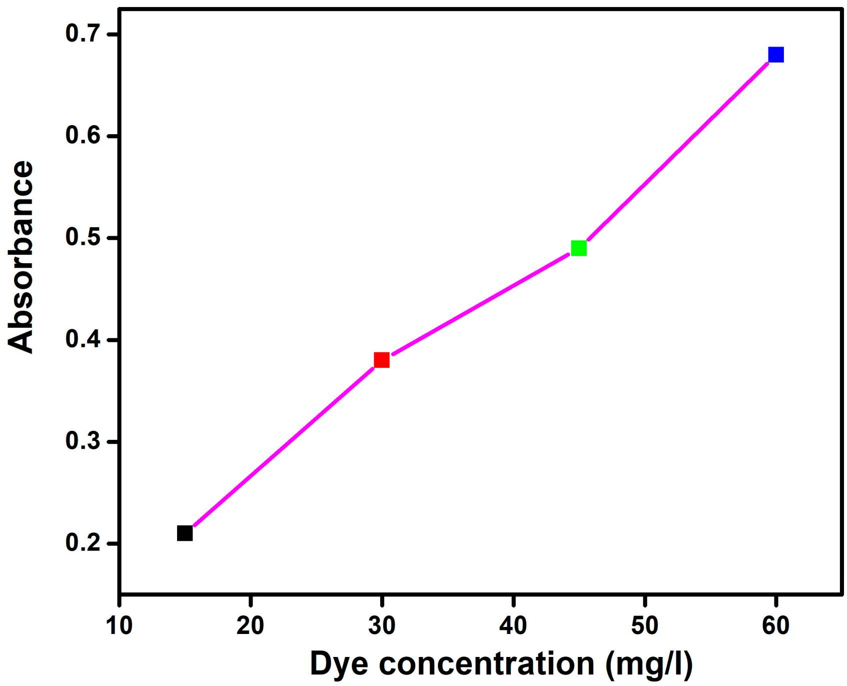

Figure S4. Variation in absorbance with dye concentration for chromium complex

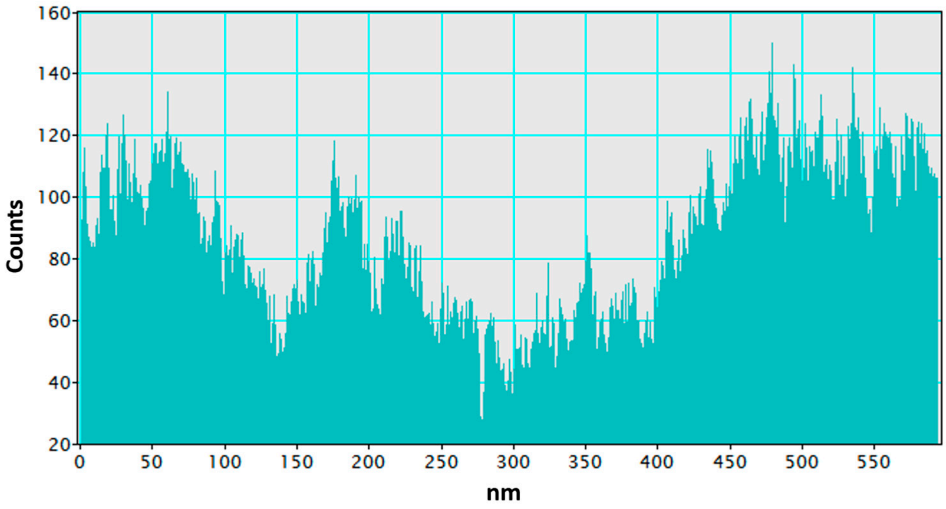

Figure S5. TEM surface profile spectrum of the CS sample

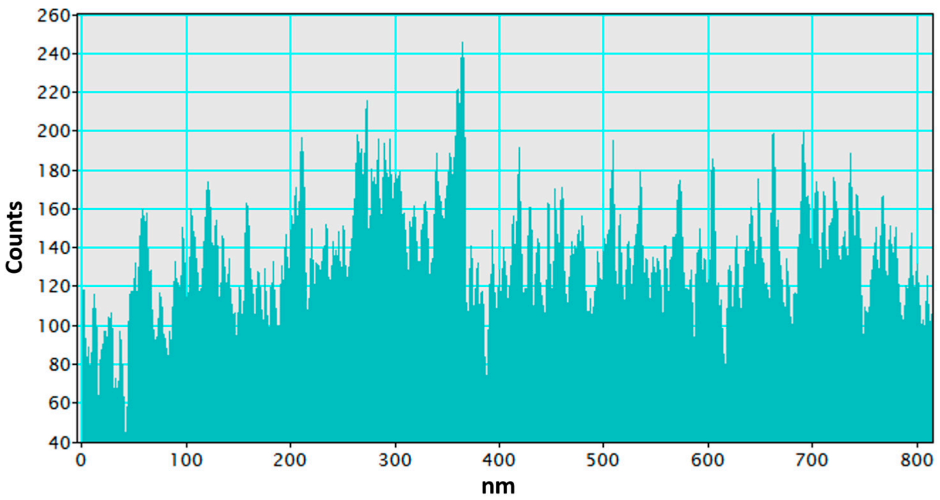

Figure S6. TEM surface profile spectrum of the CS-ZnO hybrid structure

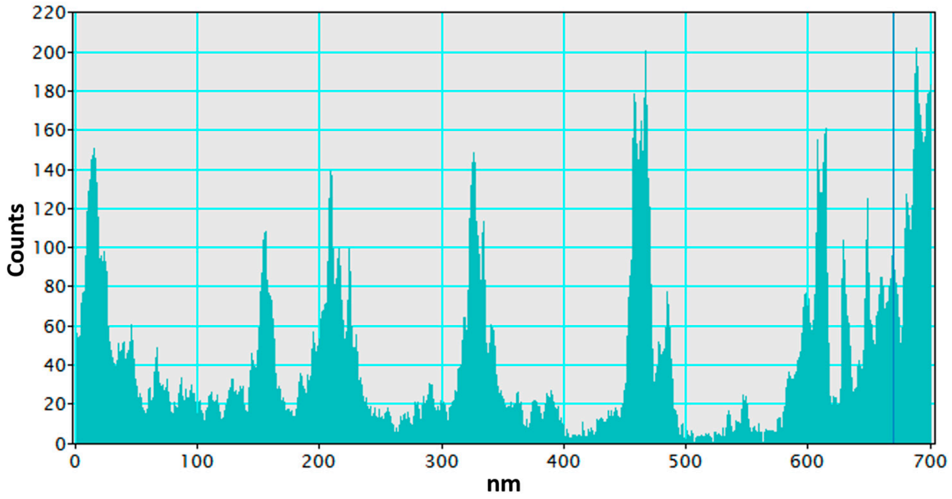

Figure S7. TEM surface profile spectrum of the CS-ZnO-GO hybrid structure
